# A Case of Eosinophilic Dermatosis of Hematologic Malignancy Treated With Narrow-Band Ultraviolet B (NBUVB)

**DOI:** 10.7759/cureus.35734

**Published:** 2023-03-03

**Authors:** Sharon Pan, Lindsay Bicknell

**Affiliations:** 1 Dermatology, Baylor Scott & White Health, Temple, USA

**Keywords:** narrow band-ultraviolet b (nb-uvb), narrow-band uv-b, insect bite-like lesion, eosinophilic dermatosis of hematologic malignancy, eosinophilia, chronic lymphocytic leukemia (cll)

## Abstract

Chronic lymphocytic leukemia (CLL) is one of the most common leukemias in adults. It has been associated with a number of dermatologic manifestations, such as leukemia cutis and erythema multiforme. Among the rarer of these findings is eosinophilic dermatosis of hematologic malignancy (EDHM). EDHM was originally characterized as a hypersensitive insect bite-like reaction, despite most patients having no distinct recollection of being bit or having any risk of exposure. Typically, EDHM presents as a pruritic, erythematous eruption, often with papulovesicular lesions, throughout the body. Due to its relapsing course, a number of treatment methods have been proposed, but no standard of care has been established. In this report, we present a recalcitrant case of EDHM in a patient with CLL that responded well to treatment with narrow-band ultraviolet B (NBUVB) light therapy.

## Introduction

Chronic lymphocytic leukemia (CLL), an adult leukemia caused by malignant proliferation of B-cells, is associated with a number of dermatological conditions, such as leukemia cutis and erythema multiforme [1,2]. Dermatologic manifestations associated with CLL may be categorized as specific (those due to direct tissue infiltration of leukemic cells) or nonspecific (those with an etiology unrelated to leukemic pathophysiology) [2]. A rare nonspecific manifestation known as eosinophilic dermatosis of hematologic malignancy (EDHM) was originally characterized as a hypersensitive insect bite-like reaction, despite most patients having no recollection of experiencing an insect bite [3-6]. While EDHM is associated with various hematologic malignancies, it occurs most commonly with CLL according to the literature. Since it was first described in 1965, a number of different descriptors have been proposed for EDHM [6]. Barzilai et al. proposed an “insect bite-like reaction” or “eosinophilic eruption of hematoproliferative disease” [4]. Other suggested names include “eosinophilic dermatosis of myeloproliferative disease” and “hematologic-related malignancy-induced eosinophilic dermatosis,” or “He Remained” [2,7-9]. The aim of this report is to describe the potential usage of narrow-band ultraviolet B (NBUVB) (NBUVB) in the treatment of EDHM.

This case report was previously presented at Texas A&M University Student Research Week on 03/24/22, Texas Dermatological Society Annual Spring Meeting on 04/29/22, Baylor Scott & White Medical Center - Temple Research Scholars Day on 05/05/22, Brigham and Women’s Hospital Dermatology Medical Student Virtual Symposium on 05/25/22. The patient provided verbal and written consent for treatment and for the publication of the report with photos.

## Case presentation

A 72-year-old female patient with a history of CLL presented with a several-week history of large, erythematous, pruritic wheals on the extremities that had appeared after she worked in the garden. At this time, although the patient had no distinct recollection of insect bites, she was diagnosed with exuberant insect bite reactions based on the belief that patients with CLL may develop significant local reactions to insect bites. The lesions resolved within a couple of months of topical steroid use as needed up to twice a day.

**Figure 1:**
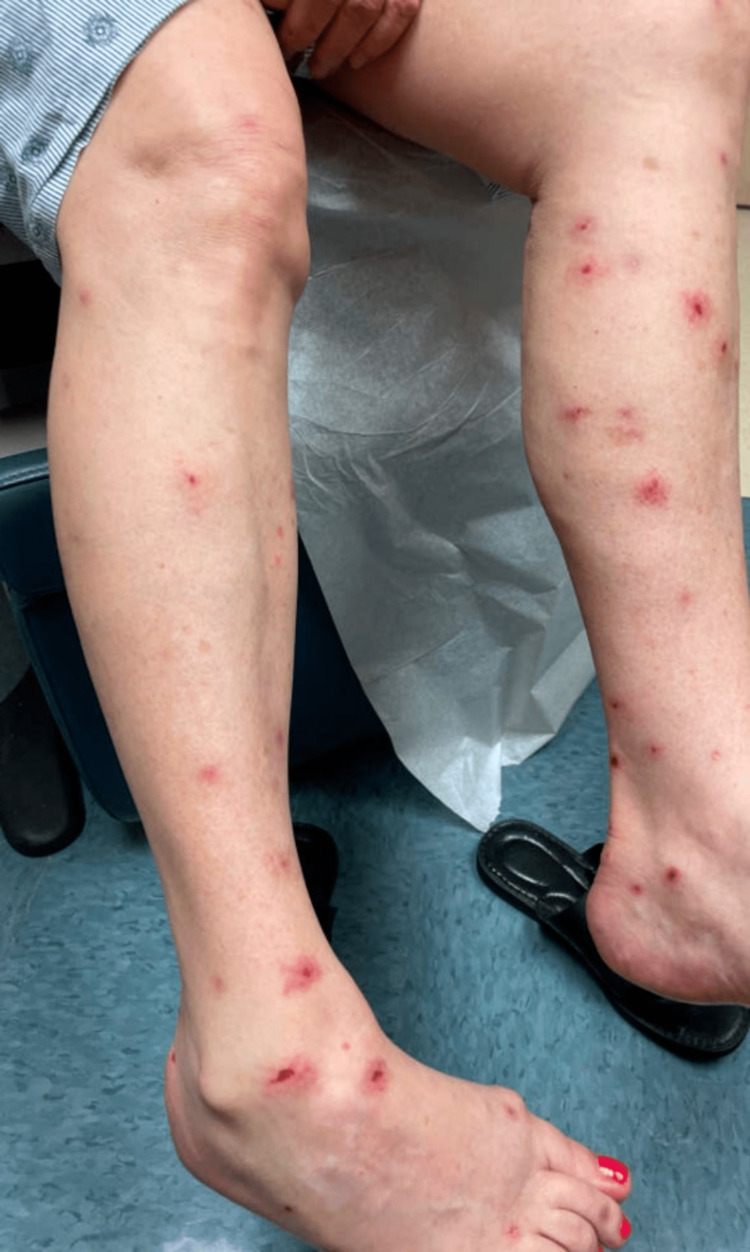
Erythematous erosions at the site of prior bullae on bilateral lower extremities

**Figure 2:**
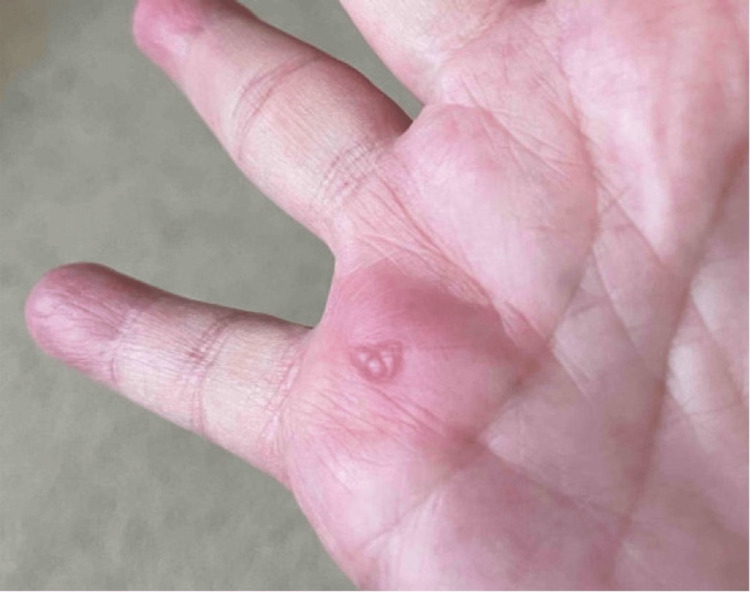
Intact vesicular lesion on the right palmar surface

Two years later, she returned to the clinic with a similar but more severe presentation of extremely pruritic, burning pink papules, wheals, and small bullae with subsequent erosions located on the extremities, palms, chin, cheeks, and forehead (Figures 1, 2). She had experienced a relapse of her CLL and was undergoing treatment with bendamustine and rituximab. Bendamustine was subsequently discontinued due to concern for a drug reaction, with no change in the development of lesions.

A biopsy of the left leg was performed and pathology results showed a small-vessel vasculitis with eosinophils, papillary dermal edema, and intraepidermal vesicles. CBC and CMP were within normal limits. Direct immunofluorescence (DIF) was not performed, but serum bullous pemphigoid antigens BP180 and BP230 were negative. Based on clinical presentation, concurrent diagnosis of CLL, and findings on the biopsy specimen, a diagnosis of EDHM was made. Therapy was initiated with topical steroids followed by oral steroids, oral doxycycline 100 mg twice daily, and nicotinamide 500 mg twice daily, as these have all been suggested to have therapeutic benefits in previous reports of EDHM [2-5,7,10-12]. However, the patient continued to develop new lesions. Additionally, she did not tolerate doxycycline well and experienced swelling with nicotinamide; hence, both were discontinued about a week later. She reported mild transient improvement with her intravenous immunoglobulin (IVIG) as prescribed by her oncologist for a separate indication.

NBUVB has been described in use for the treatment of EDHM [13] and the patient opted to initiate this therapy given unsuccessful disease control with previous therapies. The patient was then approved for an NBUVB home unit with a standard dosing regimen based on the National Biological device algorithm, appropriate for a Fitzpatrick skin type II. She started with phototherapy use three times a week, and improvement was first noted after two treatments. After one month, she reported the resolution of her lesions and ceased to develop new lesions. At that time, she reduced the phototherapy sessions to two times a week. Concurrently, the patient was approved for subcutaneous dupilumab as this has also been reported in the literature to have benefits for EDHM, but she elected not to start it since she had experienced improvement with NBUVB therapy [14]. Eventually, the patient self-discontinued the phototherapy after all her prior lesions resolved and the development of new lesions ceased. A few months later, she developed a recurrence of EDHM-like lesions on the lower extremities, which were similar in morphology to her previous biopsy-proven lesions. She restarted treatment with the home phototherapy unit and reported a similar positive response. Almost a year and a half has passed since we first used NBUVB for this patient, and while she continues to be in treatment for CLL, she has experienced no further episodes of EDHM.

## Discussion

Although EDHM is a rare entity, it may cause significant distress and detriment to patients and negatively affect their quality of life. An existing hypothesis for the pathophysiology of EDHM describes the interplay of leukemic cells with interleukin-4 (IL-4) and interleukin-5 (IL-5). These promote lymphocyte proliferation and tissue eosinophilia, which then drive the development of the lesions [5,14-16]. Another approach focuses on the contribution of underlying immunodeficiency to increased IL-5 expression and subsequent development of EDHM. This is consistent with the observation that the development of EDHM seems to be associated with more aggressive cases of CLL, as may be seen in immunocompromised patients [4,5,16,17].

EDHM most commonly occurs in patients between the ages of 40 and 60 years, and it may occur before, during, or after the diagnosis of CLL, lasting for a variable duration of time [2,11,12]. Davis et al. have reported a patient who presented with EDHM as early as approximately 10 years before the diagnosis of CLL compared to other patients in their report who developed EDHM one to seven years following the diagnosis [12]. While EDHM is associated with CLL in approximately 77% of reported cases, there are no reports of it occurring in conjunction with solid organ tumors [8,14,18]. The appearance of the lesions ranges from macular erythema and urticarial wheals to indurated nodules, papules, or vesicles [2,11,14]. To simplify the diagnosis of EDHM, Byrd et al. proposed the following diagnostic criteria: (1) pruritic, papular, nodular, and/or vesiculobullous eruption that is resistant to conservative management; (2) a superficial and deep eosinophil-rich dermal lymphohistiocytic infiltrate on pathology; (3) exclusion of other causes of tissue eosinophilia; and (4) pre-existing diagnosis of hematological malignancy [7]. Moreover, because EDHM may present with bullous lesions, direct immunofluorescence may be considered in the evaluation, but yields a negative result [17].

On pathology, EDHM demonstrates tissue eosinophilia with dermal perivascular lymphocytic infiltrate with possible spongiosis and intraepidermal vesicles that contribute to the blister-like appearance of the lesions [2,4,11,15]. Flame figures and focal collagen disruption have occasionally been reported, as well [12]. Although flame figures are associated with a variety of conditions, their presence may prompt the consideration of EDHM in the differential [19,20].

Largely due to its refractory, relapsing nature, no standard treatment regimen has been described for EDHM. Suggested treatment options include topical and systemic corticosteroids, subcutaneous dupilumab, monoclonal antibodies, IVIG, oral antibiotics, antihistamines, and phototherapy [12-14]. While systemic steroids are often the first choice, relapse is common after dose tapering [3,4]. In our case, the use of NBUVB led to the lasting resolution of prior lesions and the prevention of new lesions. Although scarring and post-inflammatory hyperpigmentation remained, this therapy offered a well-tolerated means to remission.

Limitations of this case and our hypothesis that NBUVB may be considered a safe and effective treatment option for EDHM include concurrent use of IVIG, and the possibility of spontaneous resolution (although this has not yet been reported based on our knowledge). Our patient started IVIG not long after the onset of lesions and reported only mild improvement with this treatment (which was prescribed for her CLL). Once she started NBUVB, she saw rapid improvement in current lesions and a decrease in the development of new lesions. Additionally, when her EDHM relapsed several months later, she restarted NBUVB therapy and experienced a similar positive response. This reproducibility further validates the utility of this treatment regimen.

## Conclusions

The patient described here initially presented with insect bite-like lesions and a history that corroborated a diagnosis of exuberant reaction to insect bites. She presented two years later with biopsy-proven EDHM. Treatment was attempted with topical and systemic steroids, oral antibiotics, and oral nicotinamide, with responses ranging from poor tolerance to transient improvement, at best. While she was undergoing concurrent therapy for CLL, it was only after the initiation of NBUVB that the patient experienced significant improvement in her existing lesions, with lasting resolution after the successful treatment of a second episode. Based on our experience, NBUVB may be considered as an efficacious first-line treatment for EDHM.
